# Meeting people where they are: Crowdsourcing goal-specific personalized wellness practices

**DOI:** 10.1371/journal.pdig.0000650

**Published:** 2024-11-19

**Authors:** Johanna E. Hidalgo, Julia Kim, Jordan Llorin, Kathryn Stanton, Josh Cherian, Laura Bloomfield, Mikaela Fudolig, Matthew Price, Jennifer Ha, Natalie Noble, Christopher M. Danforth, Peter Sheridan Dodds, Jason Fanning, Ryan S. McGinnis, Ellen W. McGinnis

**Affiliations:** 1 Department of Psychological Science, University of Vermont, Burlington, VT, United States of America; 2 Project LEMURS (Lived Experience Measured Using Rings), University of Vermont, Burlington, VT, United States of America; 3 Vermont Complex Systems Center, MassMutual Center of Excellence for Complex Systems and Data Science, University of Vermont, Burlington, VT, United States of America; 4 Wake Forest University School of Medicine, Winston-Salem, North Carolina, United States of America; 5 Center for Remote Health Monitoring, Wake Forest University School of Medicine, Winston-Salem, North Carolina, United States of America; 6 Department of Computer Science, University of Vermont, Burlington, VT, United States of America; 7 Department of Mathematics and Statistics, University of Vermont, Burlington, VT, United States of America; 8 Gund Institute for Environment, University of Vermont, Burlington, VT, United States of America; 9 Computational Story Lab, MassMutual Center of Excellence for Complex Systems and Data Science, University of Vermont, Burlington, VT, United States of America; 10 Department of Health and Exercise Science, Wake Forest University, Winston-Salem, NC, United States of America; Instituto Politécnico Nacional Escuela Superior de Medicina: Instituto Politecnico Nacional Escuela Superior de Medicina, MEXICO

## Abstract

**Objectives:**

Despite the development of efficacious wellness interventions, sustainable wellness behavior change remains challenging. To optimize engagement, initiating small behaviors that build upon existing practices congruent with individuals’ lifestyles may promote sustainable wellness behavior change. In this study, we crowd-sourced helpful, flexible, and engaging wellness practices to identify a list of those commonly used for improving sleep, productivity, and physical, emotional, and social wellness from participants who felt they had been successful in these dimensions.

**Method:**

We recruited a representative sample of 992 U.S. residents to survey the wellness dimensions in which they had achieved success and their specific wellness practices.

**Results:**

Responses were aggregated across demographic, health, lifestyle factors, and wellness dimension. Exploration of these data revealed that there was little overlap in preferred practices across wellness dimensions. Within wellness dimensions, preferred practices were similar across demographic factors, especially within the top 3–4 most selected practices. Interestingly, daily wellness practices differ from those typically recommended as efficacious by research studies and seem to be impacted by health status (e.g., depression, cardiovascular disease). Additionally, we developed and provide for public use a web dashboard that visualizes and enables exploration of the study results.

**Conclusions:**

Findings identify personalized, sustainable wellness practices targeted at specific wellness dimensions. Future studies could leverage tailored practices as recommendations for optimizing the development of healthier behaviors.

## Author summary

Despite many proven wellness programs, making healthy habits stick long-term remains a challenge for most people. To help overcome this, our study took a novel approach—crowdsourcing wellness practices that people already incorporate into their current lifestyles and routines. To capture these wellness practices, we gathered input from 992 individuals who felt they had been successful within specific wellness dimensions (sleep, productivity, and physical, emotional and social wellness). Intriguingly, we found little overlap in practices across different wellness dimensions, suggesting that practices are specific to dimension. Within each dimension, the most popular practices were fairly consistent across individuals, no matter their background. However, an individual’s specific health conditions did influence some of their preferred practices. For example, experiencing depression impacted which wellness practices participants gravitated towards to improve their emotional wellness. To facilitate visualization and exploration of these results, we developed an online dashboard that displays the wellness practices individuals reported as effective for improving each wellness dimension and allows users to filter by demographic, health, and lifestyle factors. Through this work, we aim to help researchers develop wellness interventions that can be tailored to each individual matched to their personal factors. By recommending wellness practices tailored to each individual, users may feel more engaged and be able to better incorporate the practices into their lifestyle and sustain them for longer.

## Introduction

Wellness has been described as the ability to function in the environment and to fulfill social responsibilities [1] and as the upper end of the spectrum of emotional and physical health [2]. Theories agree that wellness is multidimensional [3], generally including physical, emotional, social and occupational wellness with recent work validating discriminant dimensions such as sleep quality [4]. These dimensions allow for more targeted interventions for improving wellness. Maintaining wellness has proven to be challenging for many Americans. Recent data indicate concerning trends across these wellness dimensions in the United States. In terms of emotional wellness, three times as many adults report symptoms of depression or anxiety in 2024 compared to 2019 [5], and suicide rates have risen by 40% since 2000 [5]. Physical wellness has also shown concerning trends with sedentary behaviors increasing by 21% since 2001 [6], and the number of adults living with chronic pain increasing to 20% in 2016 [7]. Occupational wellness has been impacted, as evidenced by burn-out nearly doubling [8–11]. Social wellness has declined, with loneliness surging by 45% since 1979 [12,13]. Sleep has also been affected, with sleep medication use doubling to 8%, since 2010 [14,15]. These trends underscore the pressing need for effective interventions across all wellness dimensions. Luckily, wellness can be improved through the development of healthy behaviors [16]. However, often only high engagers of healthy behavior change achieve improved wellness dimensions [17–19], and rates of long-term maintenance (e.g., behavior practice for more than three months at multiple time points) of behavior change are low [20–22].

For many, cultivating wellness involves modifying a set of interrelated behaviors that vary widely in their complexity. For instance, adherence to a daily lifesaving cardiovascular medicine regimen is a relatively simple task requiring the development of a new daily habit for physical wellness. But only half of those prescribed such a medication take it regularly [23]. By contrast, modifying sedentary and dietary behaviors requires constant behavioral awareness and day-long inhibition of desirable behaviors (eating delicious food and relaxing) in the face of psychosocial stressors and changing environments. Several consistent themes have emerged from decades of behavioral sciences research focused on improving wellness via behavior change targeting specific wellness dimensions. First, health behavior adoption is a function of an individual’s preferences and prior experiences, the social and built environments in which they live, and the traits of a specific behavior of interest [24]. Second, preferences, personal needs, and environments are dynamic, and as such, behaviors can become more easy or difficult over time, and often these changes occur abruptly (e.g., due to changes in social support as in the cessation of a clinical intervention, [25]). Third, individuals are more likely to participate in activities that allow for a sense of autonomy, cultivate a sense of competency, allow for social connectivity, and are intrinsically enjoyable or satisfying [26,27]. Finally, successfully self-regulating most behaviors first requires developing an accurate awareness of current behavioral patterns [28]. For instance, behaviors tightly tied to habits (e.g., taking a new medication, reducing snacking) require raising mindless moments to mindful awareness. Adding new, effortful behaviors to one’s day often requires the use of tools to address internal behavioral monitoring. Notably, there is little correlation between recalled activity behaviors and objectively monitored activity behaviors [29] e.g., individuals underestimate calories consumed by 20–30% [30].

Several common behavior change strategies have emerged to address these behavioral requirements. For instance, those interested in intervening on relatively simple, habitual behaviors have found success emphasizing the importance of flexibly performing small healthy practices to promote self-efficacy, perceived competence, and habit formation [31]. Effective interventions targeting long-term, complex behaviors (e.g., sleep, diet, activity) focus on incremental progression to enhance feelings of competency and self-efficacy, opportunities to develop a sense of social connection, the use of self-monitoring technologies and goal setting, and perhaps most importantly, individual tailoring to preferences and barriers. Advances in digital health have allowed health providers to better address each of these dimensions by delivering awareness cues for habitual behaviors in real-time; facilitating real-time self-monitoring, feedback provision, and goal tailoring; and providing access to social connections remotely [32]. Indeed, across a number of wellness dimensions, studies have shown that tailoring intervention content and delivery to the demographics of the person, their changing preferences, and barriers is more effective than employing uniform and nonspecific intervention strategies [33–42].

While this body of work has confirmed the necessity of tailoring suggestions of which healthy behaviors to adopt to individual’s personal preferences, needs, and circumstances, practical implementations remain largely prescriptive and within specific dimensions. Indeed, although various healthy behavior recommendations are available across wellness dimensions, their adoption and effective utilization by users remain limited, highlighting a disconnect between theoretical knowledge and practical implementation. To bridge this gap, we take a broader look than existing literature, seeking to understand, across multiple wellness dimensions, what behavioral change strategies individuals adopt across demographics, health statuses, and lifestyle factors.

To this end, we recruited a representative sample of 992 Americans and asked them to choose, from a curated list, the wellness practices that help them achieve success with a specific wellness dimension. We aimed to 1) identify user-recommended wellness practices specific to sleep, productivity, physical, emotional, and social wellness and 2) examine how user recommendations differ by demographic, health, and lifestyle factors. These data, available via an open-source web dashboard, advance our knowledge of accessible, engaging wellness practices and may help to inform future personalized interventions.

## Method

### Recruitment

In 2023, 992 participants were recruited via Prolific, an online crowd-sourcing platform, to reflect the age, gender, and racial characteristics of the U.S. population (see Table 1 for participant demographics). Prolific has been shown to yield reliable and valid data [43]. In recent literature, Prolific users have demonstrated higher scores in comprehension, honesty, and attention measures when compared to users from other online data-sourcing platforms [40].

**Table 1:** Participant demographic, health, and lifestyle factors (N = 992).

Characteristic	Details	% (n) or M (SD)
Age	Range 19–76 years	(*M* = 45.77, *SD* = 15.92)
	Emerging Adult (19–29)	22% (n = 214)
	Middle Adult (30–49)	35% (n = 343)
	Older Adult (50–76)	44% (n = 433)
Racial/Ethnic Background	White	76% (752)
	Minority	24% (109)
Gender	Man	47% (446)
	Woman	49% (492)
	Other gender identity	3% (32)
Sexual Orientation	Heterosexual	82% (815)
	LGBTQ+	16% (162)
	Bisexual	9% (90)
	Other sexual orientation	3% (27)
Income	Less than $49,999	33% (333)
	$50,000+	65% (657)
Region	Northeast	20% (203)
	South	44% (447)
	Midwest	20% (196)
	West	14% (144)
Mental Health Diagnosis	Any	43% (426)
	Depression	23% (223)
Physical Health Diagnosis	Any	50% (497)
	Cardiovascular Disease	5% (47)
Use Wearable Device	Yes	41% (409)
	No	59% (581)
Use Wellness App	Yes	18% (175)
	No	82% (815)
Personality Traits	Extraversion	(M = 2.75, SD = 0.94)
	Low	54% (538)
	High	46% (452)
	Agreeableness	(M = 3.92, SD = 0.80)
	Low	42% (415)
	High	58% (575)
	Conscientiousness	(M = 3.73, SD = 0.98)
	Low	52% (514)
	High	48% (476)
	Negative Emotionality	(M = 2.65, SD = 1.15)
	Low	57% (560)
	High	43% (430)
	Open-Mindedness	(M = 3.92, SD = 0.84)
	Low	41% (440)
	High	59% (585)
Short Self-Regulation Scale	Self-regulation	
	Low	44% (439)
	High	55% (551)
	Goal setting	(M = 22.35, SD = 4.98)
	Perseverance	(M = 10.34, SD = 2.76)
	Decision making	(M = 16.39, SD = 5.17)
	Learning from mistakes	(M = 11.67, SD = 2.43)

*Note*. Numbers within categories may not add up to presented N due to missing values or preference not to respond.

### Design

A list of 50 practices for improving sleep, productivity, and physical, social, and emotional wellness was compiled based on literature reviews of efficacious wellness interventions e.g. [44–50], recommendations from the National Institutes of Health (NIH) webpage “Your Healthiest Self: Emotional Wellness Toolkits”, and feedback from a development phase. An initial “development” survey was deployed to 300 Prolific participants representative of the U.S. population. Participants were asked to select 3 of the 5 wellness dimensions they felt they excelled the most. For each of their three selected dimensions, participants were asked to choose 3–5 practices they regularly engage in that most helped them improve that wellness dimension from a randomly ordered list of the 50 curated wellness practices with 5 additional write-in options. The “development” survey aimed to refine the list of popular wellness practices and inform the development of the Wellness Dimension Survey used in the current study. The Wellness Dimension Survey included the top 25 wellness practices engaged in by wellness dimension from the “development” survey results. Participants were compensated $4 for study completion.

### Measures

The Wellness Dimension Survey began with the item, “We want to help people struggling to accomplish wellness goals learn from you! Choose 3 wellness dimensions you’re doing best at, so we can learn what works for you!”. Wellness dimensions included sleep, and physical, emotional, and social wellness as well as a piece of occupational wellness which we operationalized as productivity. Participants were then instructed to select three to five items from a curated list of 25 wellness practices specific to each wellness dimension (with 3 additional write-in options) that they engage in regularly to help improve their wellness dimension. For each chosen practice, they were asked the ideal duration and number of days per week they would practice to best improve their dimension. In addition to predetermined options, participants were given the opportunity to provide write-in responses in the Wellness Dimension Survey. These write-in responses were analyzed for recurring themes, and the frequency of each theme was tallied. Our analysis revealed that no single theme from these write-in responses appeared in more than 4% of the total responses. This low frequency suggests that the predetermined options in the survey adequately captured the majority of participants’ perspectives on wellness dimensions. After participants selected their top wellness practices, they were prompted to provide a personal narrative about how they have incorporated those practices into their daily lives and how they benefited from them. Although the question was not mandatory, 63% (n = 623) of participants responded. Answers typically ranged from a sentence to a paragraph in length and focused on one or two practices.

To better understand what impacts wellness practices, participants were assessed on a variety of demographic, health, and lifestyle factors. Demographic assessments included age, gender, sexual orientation, ethnicity, employment status, income, and location. Health questions inquired about current physical and mental health diagnoses. Other lifestyle factors included assessing the use of wearables and wellness apps, personality traits, and regulation of behaviors. Personality traits of participants were measured with the Big Five Inventory-2-Extra Short Form (BFI-2-XS) [51], consisting of 15 five-point Likert scale items. The BFI-2-XS internal consistency ranged from 0.58 to 0.79 across subscales: Extraversion (*α* = 0.60), Agreeableness (*α* = 0.58), Conscientiousness (*α* = 0.70), Negative Emotionality (*α* = 0.79), Open-Mindedness (*α* = 0.65). Regulation of behaviors to achieve targeted objectives was measured with the Short Self-Regulation Questionnaire (SSRQ) [52] consisting of 17 five-point Likert scale items. The SSRQ demonstrated excellent internal consistency for the total questionnaire items (*α* = 0.93). Good internal consistency was established for the factors Goal Setting (*α* = 0.89), Decision Making (*α* = 0.88), and Learning from Mistakes (*α* = 0.80), and Perseverance (*α* = 0.76). Data analyses were conducted using IBM SPSS Statistics Version 29 and Python 3.12.1.

### Analytic plan

We present descriptive statistics on the top 5 of 25 wellness practices selected within each wellness dimension in rank-ordered lists and indicate their ideal duration and weekly frequency as detailed by participants who self-identify as engaging successfully in that practice. We also present descriptive statistics on the most frequently chosen wellness dimensions and evaluate selected group differences by demographic, health, and lifestyle factors using chi squared tests. We compare preferred wellness practices by demographic, health, and lifestyle factors using Rank Biased Overlap (RBO), which quantifies the similarity between rank-ordered lists [53]. An RBO of 0 indicates disjointed lists and an RBO of 1 indicates identical lists. Lastly, we provide case studies highlighting relationships between demographic, health, and lifestyle factors and wellness practices within the wellness dimensions.

## Results

Nine hundred ninety-two participants completed the survey **(Table 1)**. Most participants were White, non-Hispanic (76%), had completed at least a bachelor’s degree (56%), held at least part-time employment (68%), and lived in suburban areas (41%).

### Top wellness practices by dimension

Participants selected three of the five wellness dimensions (sleep, productivity, physical, emotional, and/or social) in which they excelled **(Table 1)**. The top wellness practices for each dimension are reported in **Table 2**, along with the ideal recommended duration and weekly frequency. Complete ranked lists of all 25 response options by wellness dimension are available in the **Supplementary Material (**S1 Table).

**Table 2:** Frequencies of top 5 practices by wellness dimension (N = 992).

Wellness Dimension (Frequency)	Health Practices	Frequency Chosen	Ideal Duration in Minutes	Ideal Days/ Week
Sleep (65% (n = 649))	Sleep in a dark, quiet environment	55.2% (358)	NA	6.5 (1.5)
	Stick to consistent sleep/wake times	54.2% (353)	NA	5.9 (1.7)
	Limit caffeine consumption	42.5% (276)	NA	5.8 (2.1)
	Stick to a nightly routine	31.3% (203)	NA	6.1 (1.5)
	Avoid naps	26.2% (170)	NA	5.5 (2.3)
Physical* (67% (n = 661))	Drink plenty of water	59.0% (385)	33.6 (48.0)	6.6 (1.3)
	Exercise moderately (run, bike, weights)	52.5% (342)	52.8 (30.7)	4.6 (1.7)
	Exercise lightly (walk, dance)	33.9% (221)	46.4 (26.1)	5.2 (1.7)
	Stick to consistent sleep/wake times	25.2% (164)	91.2(166.3)	6.0 (1.7)
	Play with your pets	24.8% (162)	47.2 (38.2)	6.3 (1.3)
Emotional (66% (n = 652))	Watch a TV show, movie, or read to relax	50.5% (334)	102 (68.6)	6.0 (1.6)
	Listen to music	46.7% (309)	86.4 (79.2)	5.6 (1.6)
	Play with your pets	35.7% (236)	50.6 (40.2)	6.4 (1.3)
	Spend in-person time with loved ones	33.0% (218)	132.6 (108.6)	5.4 (2.1)
	Spend time in nature	32.2% (213)	53.2 (33.3)	4.3 (2)
Productivity (61% (n = 605))	Write down a list of your priorities	42.5% (257)	16.3 (13.2)	4.8 (1.9)
	Create a schedule or plan	41.0% (248)	22.1 (20.7)	5.0 (2)
	Schedule time for uninterrupted work	31.6% (191)	120.4 (128.6)	5.0 (1.4)
	Tackle a task on your to-do list	29.4% (178)	58.7 (69.1)	5.2 (1.7)
	Stick to consistent sleep/wake times	28.8% (174)	72 (144.6)	6.0 (1.6)
Social (41% (n = 409))	Spend in-person time with loved ones	55.0% (225)	127.8 (99.0)	4.4 (2.4)
	Text/call a loved one or friend	52.6% (215)	44.4 (49.2)	4.9 (2.0)
	Share a meal with a friend or family	42.1% (172)	59.3 (35.9)	4.3 (2.4)
	Join a group focused on a favorite hobby	25.9% (106)	69.3 (52.1)	2.6 (2.0)
	Distance yourself from someone toxic	16.4% (67)	13.8 (23.7)	4.1 (2.8)

*Note: Eating a healthy meal was omitted from the option list in error and may have otherwise been present in the top practices for Physical Health as indicated by 32 write-ins with this theme.

### Differences by demographic, health, and lifestyle factors

**Fig 1:**
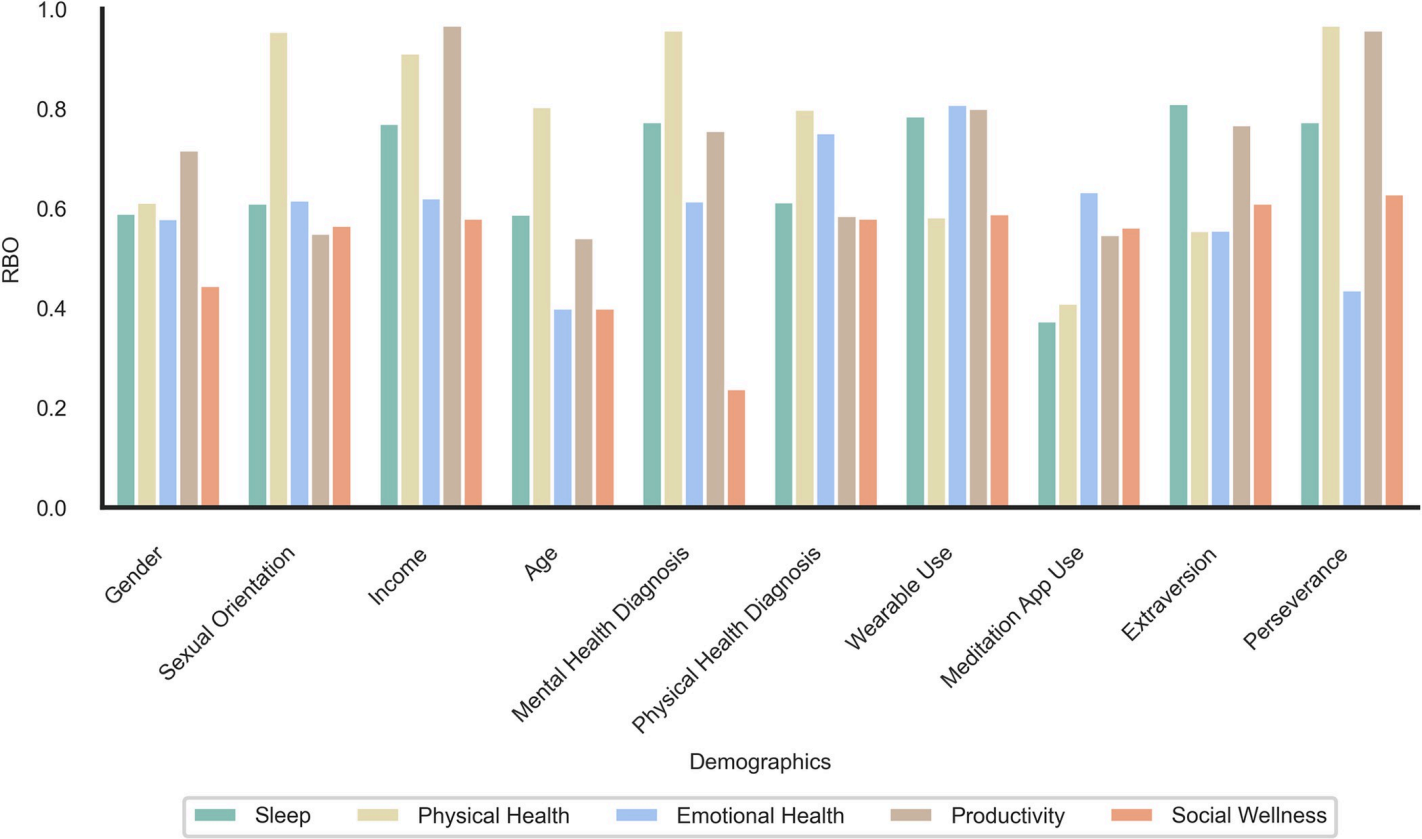
Percentage difference between individuals who chose health goals by dichotomous demographic, health, and lifestyle factors. Gender (men—women); Sexual Orientation (heterosexual—sexual minority); Income (family earnings < $50,000—family earnings > $50,000); Age (17–30–50–76); Mental Health (MH) Diagnosis (At least one mental health diagnosis—none); Physical Health (PH) Diagnosis (At least one physical health diagnosis—none); Wearable Use (Wears a wearable device—does not); Meditation App Use (Uses Calm or Headspace Apps—not); Extraversion (High—Low; cut at mean); Perseverance (high–low; cut at mean).

**Fig 1** indicates the difference in percentage between participant subgroups within several example demographic, health, and lifestyle groups who report excelling in each of the five wellness dimensions. Comparisons are not exhaustive but represent demographic and lifestyle factors of interest. Among participants with a physical health diagnosis, fewer reported excelling within the physical wellness dimension (57%, n = 288) than those without such a diagnosis (74%, n = 364) (x^2^(*1*) = 27.78, *p* < .001). Interestingly, those with a mental health diagnosis reported excelling within the emotional wellness dimension (65%, n = 277) at similar rates to those without a mental health diagnosis (68%, n = 383) (x^2^(*1*) = .909, *p* = .340). Those with wellness apps (mostly Headspace or Calm) chose sleep as a wellness dimension they excelled in less frequently (57%, n = 99) than those without wellness apps (67%, n = 548) (x^2^(*1*) = 7.24, *p* = .007). Those who earn more than $50k annually indicated that they excelled at productivity at a similar rate (62%, n = 406) to those who earn less than $50k annually (60%, n = 198) (x^2^(*1*) = 0.50, *p* = .476). Those who scored as high extraversion indicated excelling at social wellness at a similar rate (48%, n = 68) to those who scored as low extraversion (40%, n = 339) (x^2^(*1*) = 2.86, *p* = .091).

**Fig 2:**
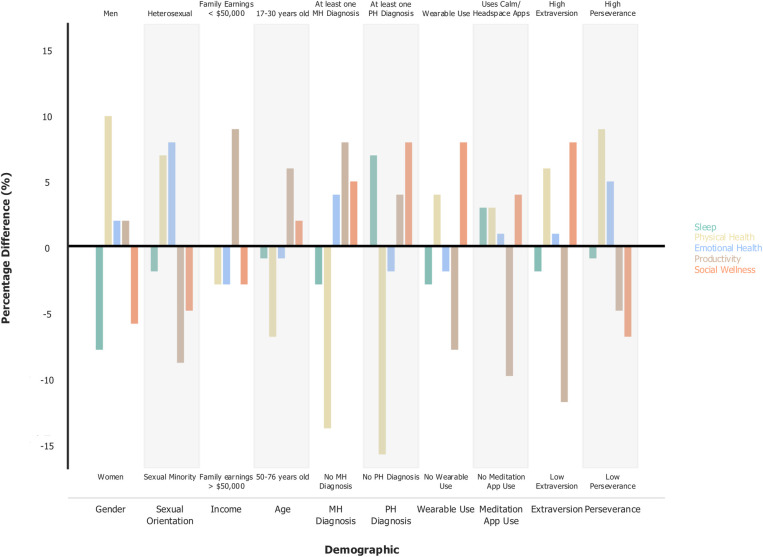
Rank biased overlap scores for each health goal across demographics. Gender (woman vs man); Sexual Orientation (heterosexual vs sexual minority); Income (family earnings < vs > $50,000); Age (17–30 vs 50–76); Mental Health (MH) Diagnosis (At least one mental health diagnosis vs none); Physical Health (PH) Diagnosis (At least one physical health diagnosis vs none); Wearable Use (Wears a wearable device vs does not); Meditation App Use (Uses Calm or Headspace Apps vs not); Extraversion (High vs Low cut at mean); Perseverance (high vs low cut at mean). RBO is a similarity measure that falls between 0–1, where an RBO of 0 means the lists are disjoint, and an RBO of 1 means the lists are identical.

To understand how top practices varied by demographic, health, and lifestyle factors we calculated the RBO score between the top 10 wellness practices lists **(Fig 2)**. Comparisons are not exhaustive but represent demographic and lifestyle factors of interest, with group sizes of at least 10% of the overall sample. More granular comparisons are illustrated in supplemental material and can be recreated on the dashboard (described in the Dashboard section).

Ranked lists of wellness practices are relatively similar (RBOs ranged between 0.62–0.99) amongst demographic comparisons (e.g., gender, sexual orientation, income, age, and location), the presence of a physical or mental health diagnosis, and lifestyle factors (using a wearable device or wellness app) across wellness dimensions. Much of the difference between lists occurs in less popular practices, as the most common 3–4 practices are generally identical. Across factors, physical wellness and productivity practices are the most similar, with RBO scores largely above 0.80. Conversely, practices for social wellness are the most dissimilar across demographic, health, and lifestyle factor splits, with only the practices split by sexual orientation resulting in an RBO score greater than 0.80.

#### Case studies to highlight similarities and differences in wellness practices

**Fig 3:**
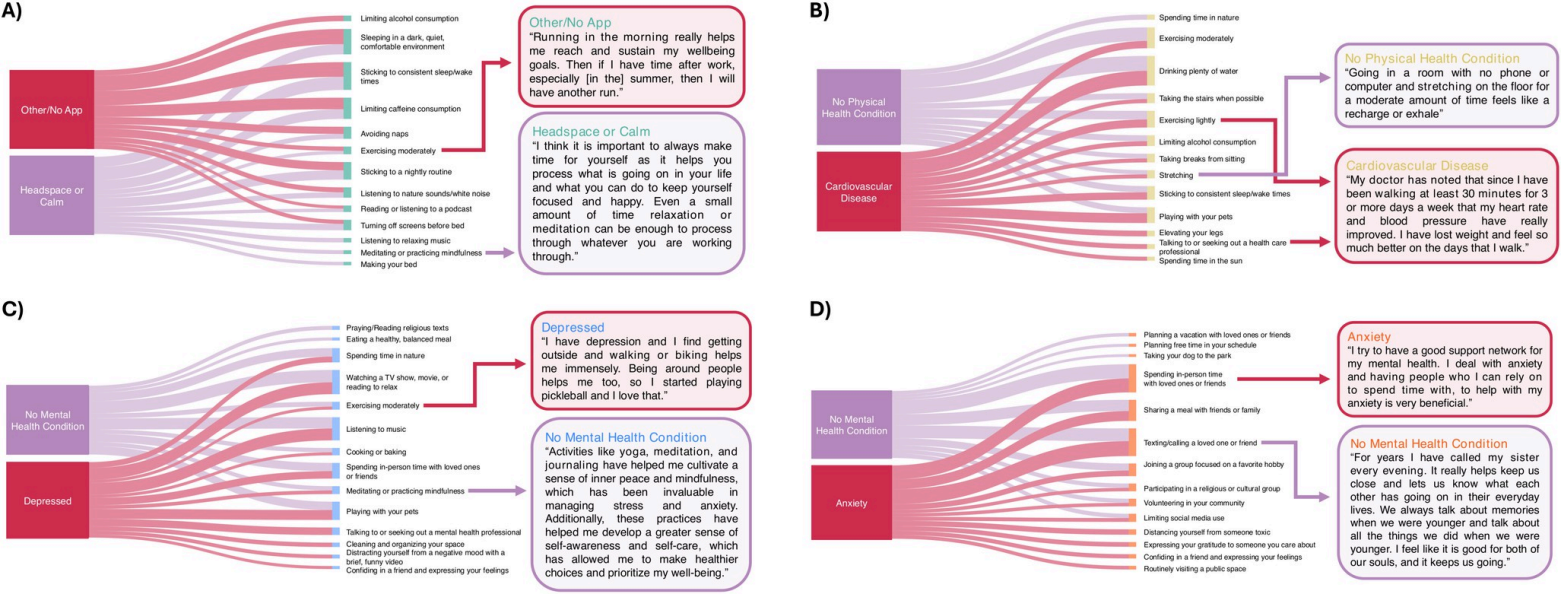
Sankey diagrams. (A) Top ten practices for improving sleep as preferred by participants with and without the Headspace or Calm apps. (B) Top ten practices for improving physical health as preferred by participants with and without cardiovascular disease. (C) Top ten practices for improving emotional health as preferred by participants with depression compared to those without any mental health condition. (D) Top ten practices for improving social wellness as preferred by participants with anxiety compared to those without any mental health conditions.

The Sankey diagrams in **Fig 3** highlight several case studies of the similarity between top wellness practices within dimensions by demographic, health, or lifestyle factors. We have also included example personal narratives from several participants about how they have incorporated those practices into their daily lives and how they benefited from them. Readers interested in further comparisons can explore them across all wellness dimensions on the dashboard. For sleep, we compare individuals owning the Headspace or Calm wellness apps to those who do not (RBO = 0.69). Owning one of these apps that target mindfulness practice is associated with the practice of “meditating and practicing mindfulness” for improving sleep, but this is not true for those without these apps who instead prioritize “limiting alcohol consumption.” For physical wellness, we compare individuals with cardiovascular disease to those without (RBO = 0.79). Having a cardiovascular condition is associated with the practices of “elevating your legs,” “talking to or seeking out a healthcare professional,” and “spending time in the sun,” whereas those without a physical health condition were more likely to focus on “spending time in nature” to improve their physical wellness. For emotional wellness, we compare individuals with depression to those without a mental health condition (RBO = 0.71). Having depression is associated with practices such as “talking to a mental health care professional,” “cleaning,” “watching a funny video,” and “confiding in a friend,” but this is not true for those without depression who were more likely to “pray” and “eat a healthy, balanced meal” to improve emotional wellness. For social health, we compare individuals with anxiety to those without a mental health condition (RBO = 0.62). Having anxiety is associated with practices such as “distancing yourself from someone toxic,” “expressing gratitude,” “confiding in a friend,” and “routinely visiting a public space.” In contrast, the practices of “planning a vacation,” “taking your dogs to the park,” and “planning free time in your schedule” are more common for improving social wellness for individuals without a mental health condition.

**Fig 4:**
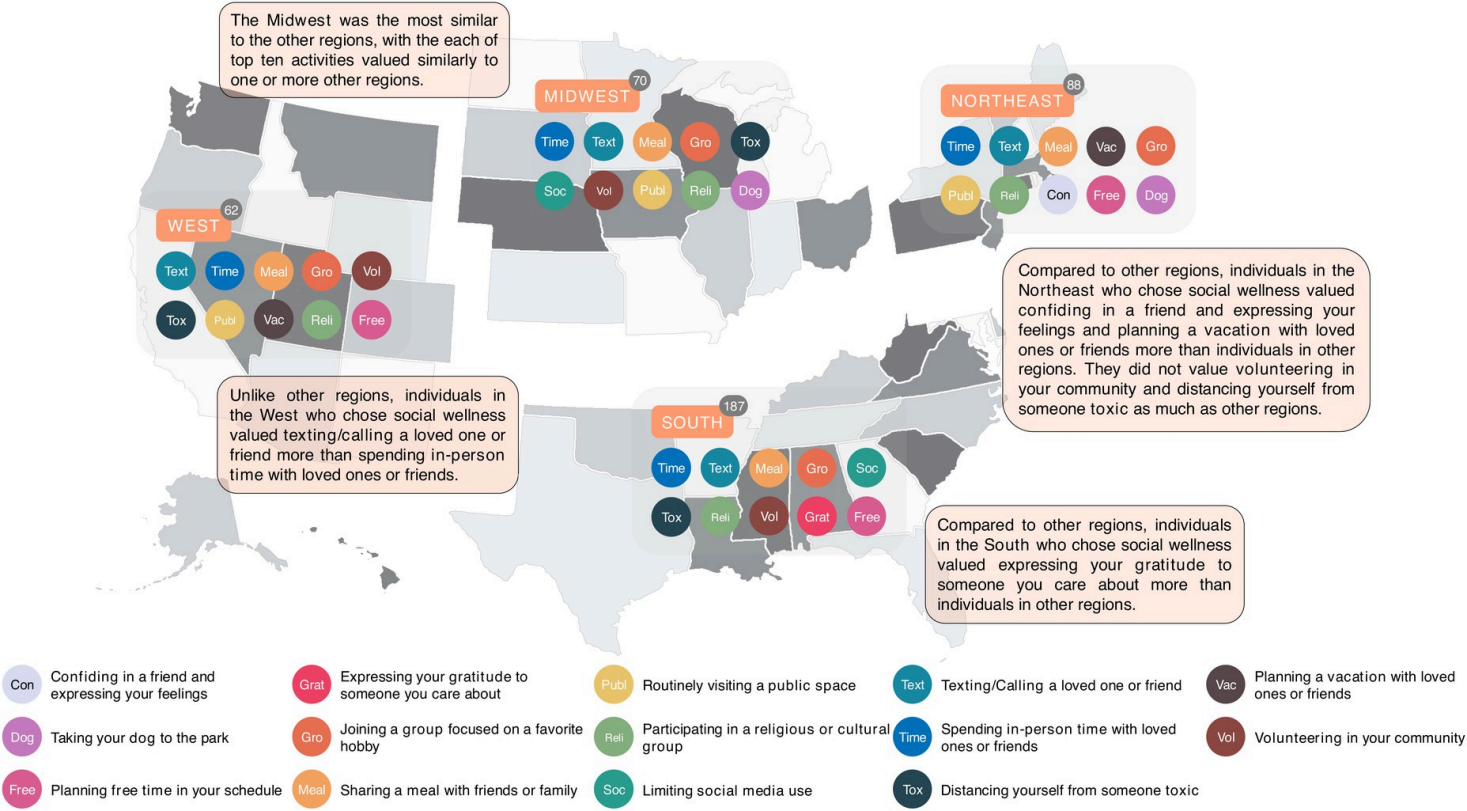
Top ten practices for social wellness by region of the United States. The base layer map of the United States depicted in this figure is adapted from an open source freely available at https://www.fla-shop.com/svg/usa/, which permits use for both commercial and personal purposes with proper attribution, under the Creative Commons Attribution 4.0 International License (https://creativecommons.org/licenses/by/4.0/).

As shown in **Fig 4**, social wellness practices varied by region within the United States. Individuals living in the northeast had two practices absent from any of the other regions (“planning a vacation with loved ones or friends” and “confiding in a friend and expressing your feelings”). Notably, the South valued “expressing your gratitude to someone you care about” more than other regions, where that practice did not make it into the top ten.

### Dashboard

**Fig 5:**
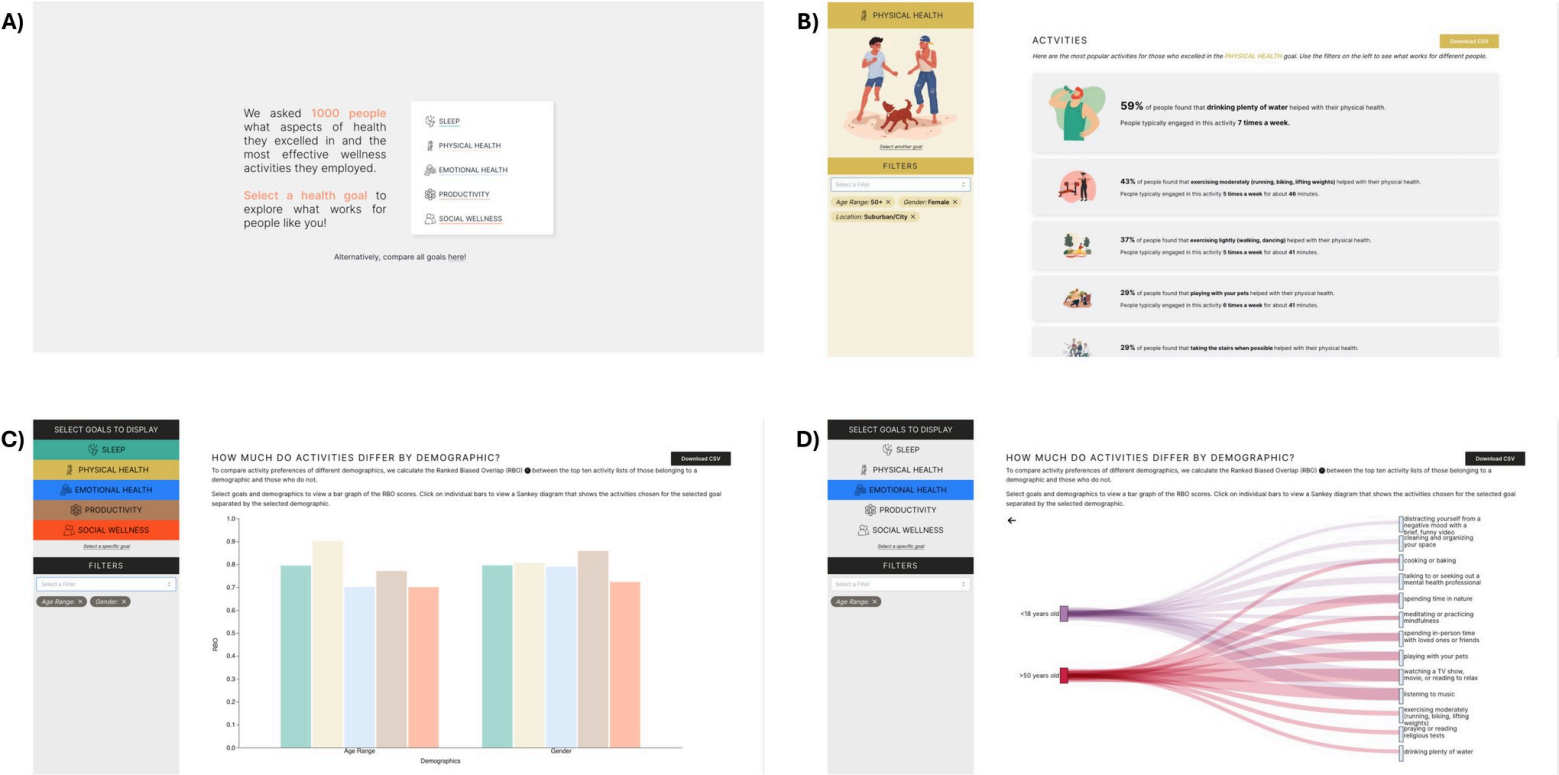
Dashboard screenshots. (A) The landing page for the dashboard allows users to select the dimension they are interested in exploring further. (B) Upon selecting a health goal, users will see the top 10 practices for that particular health goal which they can filter by demographic by selecting filters from the dropdown on the left side of the screen. In the top right corner is a “Download CSV” button that allows users to download the raw data that was used to identify the top practices. (C) By choosing to compare all goals on the landing page, users can select goals and filters to view a bar graph of the RBO scores. (D) Users can view a Sankey diagram of the wellness practices chosen for a specific goal separated by a particular demographic, health, or lifestyle factor by clicking on a specific bar in the bar graph. The images and clip-art were created using both free and Pro elements from Canva (https://www.canva.com/). All elements are used in accordance with Canva’s Content License Agreement (https://www.canva.com/policies/content-license-agreement/).

To facilitate the exploration of data, we developed a dashboard **(Fig 5)** that allows researchers, practitioners, and other interested readers to select a wellness dimension and view the top 10 practices for that dimension filtered by demographic, health, and lifestyle factors. Additionally, users can download the raw data as a CSV file for their analysis. We envision this tool being used by other researchers and practitioners interested in learning more about preferred wellness practices for sleep, productivity, and physical, emotional, and social wellness. The dashboard was developed in Next.js and can be accessed here: https://wellness-practices.vercel.app/. The images and clip-art within the dashboard were created using both free and Pro elements from Canva (https://www.canva.com/). All elements are used in accordance with Canva’s Content License Agreement for free elements and Canva’s Pro Content License Agreement for Pro elements (https://www.canva.com/policies/content-license-agreement/).

## Discussion

We asked a representative sample of 992 Americans about their wellness practices to create a crowd-sourced list of recommendations for improving sleep, productivity, physical, emotional, and social wellness. These data have been made available in a web dashboard, allowing the public to view practice recommendations by wellness dimension and demographic, health, and lifestyle factors that study participants choose to engage in. The aim of our investigation is to provide brief, flexible wellness programming recommendations personalized to user health goals and characteristics, which may be considered in future interventions.

The perceived ability to excel was equal across wellness dimensions except for social wellness. This relatively lower frequency of selection may be due to the challenges of social interactions from the pandemic [54], or reflective of the rise in loneliness [55], and may be an important wellness focus for future interventions [56]. The perceived ability to excel in specific wellness dimensions also appears to be associated with certain demographic, health, and lifestyle factors **(Fig 1)**. For subgroups who feel they are not excelling in a particular wellness dimension (e.g., those with a physical health diagnosis in physical wellness), it may be important to inform wellness interventions from those in the subgroup who do feel they are excelling to suggest practices that may work best and are sustainable. It is also important to evaluate researcher assumptions about individual needs (e.g., that those with a mental health diagnosis are less likely to excel in emotional health) based on demographic, health, and lifestyle factors.

Comparing wellness practice preferences across wellness dimensions, we found some overlap in the top ten practice recommendations. For instance, playing with pets, exercising moderately, limiting alcohol consumption, spending in-person time with loved ones or friends, spending time in nature, and sticking to consistent sleep/wake times were listed as top choices for helping to improve wellness in more than one dimension. Given their simplicity and overarching reach, these practices facilitate cost-effective and scalable options for general “wellness improvement.” Notably, the rest of the 44 top ten recommended practices were specific to a wellness dimension, pointing to the importance of making specific and conscious health goals before engaging in wellness programming. Interestingly, some well-studied evidence-based wellness programs such as meditation [57,58] and yoga [59,60] made only one wellness dimension 25-option list and no top ten practice lists. Studies employing such interventions less commonly engaged in by users may benefit from psychoeducation and/or habit formation tools such as “piggybacking” a new health behavior with an existing health practice at the onset of the treatment to elicit cues [59,60].

The selection of top practices supports current research in wellness dimensions and can help inform researchers and health providers in targeting behavior change. For instance, the top five practices for improving sleep involved consistency in the bedtime routine, supported by prior works [61]. The top practice for improving emotional wellness was relaxation, consistent with previous pandemic research [62]. Although health professionals may not typically recommend extended TV watching (≥2.5 hours, 7 days a week) [63], the theme of relaxation for emotional wellness is reflective of the increasing reports of burnout in people’s lives [64]. The top practice for physical wellness was drinking water, which suggests that feeling physically good is not restricted only to exercise, consistent with research [65]. For productivity, top practices emphasized the need for breaks and balance, consistent with literature suggesting they are essential to preventing burnout [66]. For social wellness, top practices highlighted spending time with loved ones, consistent with reports that Americans generally prefer spending time with family over other socially curated events of life [67].

In general, individuals consistently endorsed similar health practices across health goals, as evidenced by relatively high RBO scores, with the top practices being nearly identical across demographic and lifestyle groups. Despite group practice similarities, the potential impact of minor personalized adjustments on enhancing behavioral health practices and longevity cannot be underestimated. Importantly, recommendations from individuals with lived experience can serve as catalysts in effecting such minute positive changes [68–72]. Clinical providers might be interested in using the sorting system available on our dashboard to identify practices that best fit the needs of their seen population. Future research on behavior interventions might find it beneficial to select practices already being performed to optimize the sustainability of new health behaviors.

Our study has several limitations of note. First, a significant limitation of our study was the insufficient representation of diverse subgroups, particularly non-binary individuals and racial and ethnic groups. The small sample sizes within these subgroups precluded meaningful analyses of their unique experiences and intersectionality. Specifically, we did not conduct analyses of racial and ethnic differences due to inadequate statistical power, which could have led to unreliable conclusions. To address these limitations, future studies should prioritize recruiting larger, more diverse samples. This approach would enable robust examination of factors such as gender identity and racial/ethnic differences, both within individual wellness dimensions and comparatively across the entire wellness spectrum. Secondly, participants were required to select three health dimensions, which may have led some to choose dimensions they did not perceive themselves as excelling in. Future studies could allow for more open-ended dimension identification. Third, wellness practices were self-reported as beneficial and may be biased, thus future research should investigate long-term objective behavior change and corresponding wellness dimensions. Additionally, the wellness practice option order was not randomized thus, option listing may be a confounding variable. Other limitations included an erroneous omission of ’eating a healthy diet’ from the curated physical wellness dimension practices list, despite being a top-selected option in the development survey, and omission of cost-of-living adjustments for salaries in the demographics survey. Finally, it is important to emphasize that the practices represent self-reported preferences and are not recommendations from health professionals.

## Conclusion

In this work we build on this conclusion, aiming to identify wellness practices across five wellness dimensions and explore how these differ by demographic, health, and lifestyle factors. To that end, we conducted a survey with 992 individuals and explored commonalities and differences in preferred practices. Our findings suggest practices should be tailored to the dimension and individual. This personalized approach increases the probability of fostering sustainable behavior change and can inform digital interventions and the use of mHealth apps that currently have traction with users to adapt interventions [73,74]. Utilization of behavior strategies that include self-determination techniques has rarely been present in health apps for smartphones, and there is a need to further investigate its effect on adherence [22,75].

## Supporting information

S1 Fig **RBO scores for each health goal across physical (a) and mental health (b) diagnoses**. The sample size for groups with the diagnosis is displayed over each bar. Sample sizes ranged between 16–214 individuals for individual diagnoses, often representing fewer than 10% of the overall sample.
(TIF)

S1 Table Wellness Practice Options by Wellness Dimension on Phase 2 Survey Curated from Phase 1 Results. (DOCX)
