# Supplementary figures and images for: Meeting people where they are: Crowdsourcing goal-specific personalized wellness practices
Source: PLOS Digit Health. 2024 Nov 19;3(11):e0000650. doi: 10.1371/journal.pdig.0000650 (PMC11575832; doi:10.1371/journal.pdig.0000650)

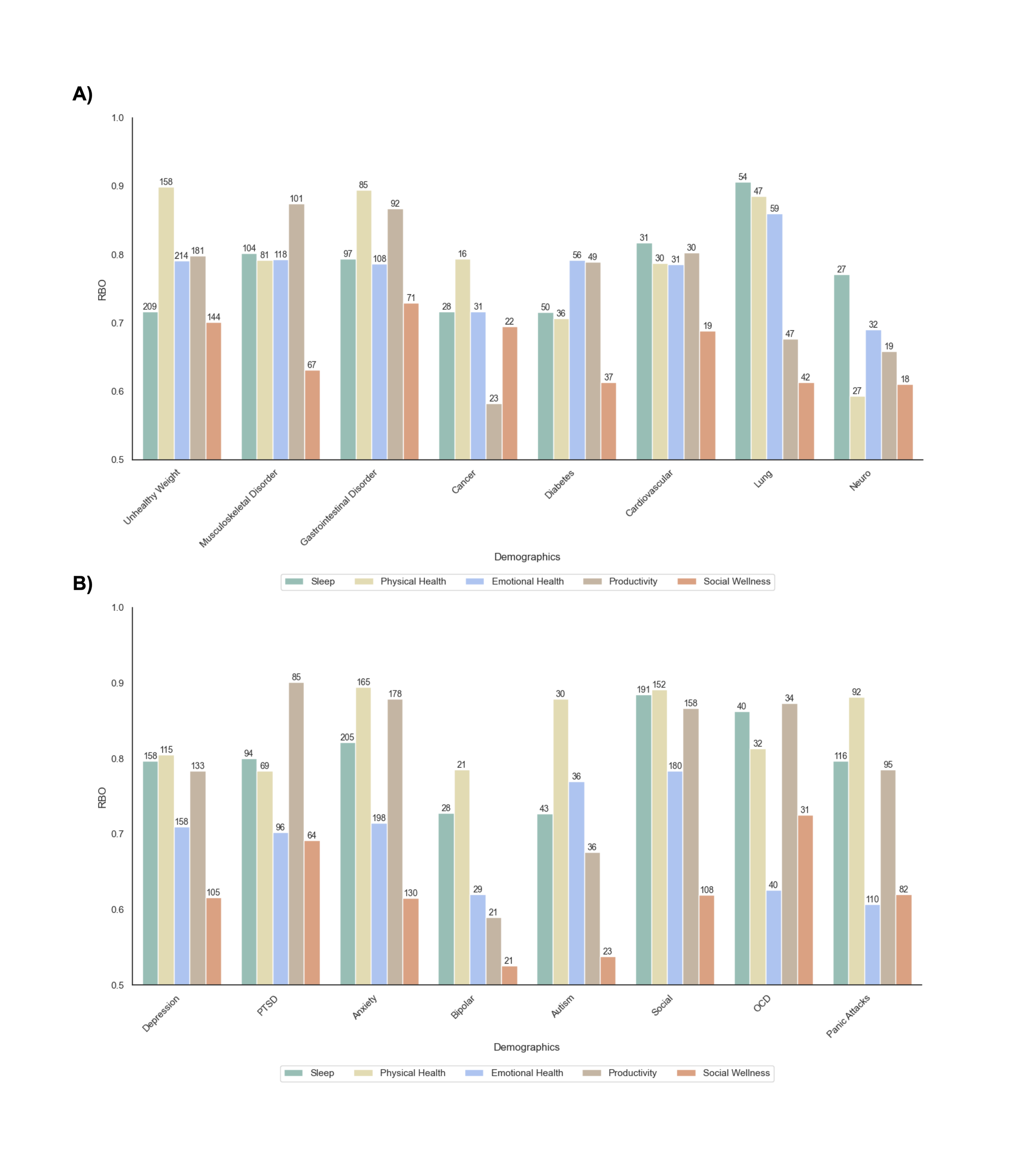

Supplement: S1 Fig — RBO scores for each health goal across physical (a) and mental health (b) diagnoses. The sample size for groups with the diagnosis is displayed over each bar. Sample sizes ranged between 16–214 individuals for individual diagnoses, often representing fewer than 10% of the overall sample. (TIF) [file pdig.0000650.s001.tif]
