# Supplementary material for: Meeting people where they are: Crowdsourcing goal-specific personalized wellness practices
Source: PLOS Digit Health. 2024 Nov 19;3(11):e0000650. doi: 10.1371/journal.pdig.0000650 (PMC11575832; doi:10.1371/journal.pdig.0000650)
Supplement: S1 Table — (DOCX) [file pdig.0000650.s002.docx]

**S1 Table. Wellness Practice Options by Wellness Domain on Phase 2 Survey Curated from Phase 1 Results**

| Sleep | Physical | Emotional | Productivity | Social |
| --- | --- | --- | --- | --- |
| Limit alcohol consumption | Play with your pets | Cook or bake | Stick to consistent sleep/wake times | Join a group focused on a favorite hobby |
| Limit caffeine consumption | Stick to consistent sleep/wake times | Talk to or seek out a mental health professional | Put your phone away/on ‘Do Not Disturb’ | Participate in a religious or cultural group |
| Avoid naps | Elevate your legs | Watch a TV show, movie, or read to relax | Create a silent environment | Volunteer in your community |
| Stick to consistent sleep/wake times | Talk to or seek out a health care professional | Volunteer in your community | Eliminate less important tasks and commitments from your schedule | Text/call a loved one or friend |
| Stick to a nightly routine | Spend in-person time with loved ones or friends | Play with your pets | Schedule time for uninterrupted work | Spend in-person time with loved ones or friends |
| Sleep in a dark, quiet, comfortable environment | Limit alcohol consumption | Listen to music | Make your bed to start your day | Share a meal with friend or family |
| Create a schedule or plan for the next day | Take the stairs when possible | Spend time in nature | Ask for help from your support network | Ask for help from your support network |
| Meditate/practice mindfulness | Drink plenty of water | Distract yourself from a negative mood with a brief, funny video | Schedule regular meetings with your coworkers | Distance yourself from someone toxic (unfollow them on social media, say no to an invite) |
| Listen to nature sounds or white noise | Exercise moderately (run, bike, lift weights) | Spend in-person time with loved ones or friends | Write down a list of your priorities | Greet and introduce yourself to strangers |
| Take a restorative shower or bath | Cook or bake | Exercise moderately (run, bike, lift weights) | Create a schedule or plan | Take your dog to the park |
| Exercise moderately (run, bike, lift weights) | Practice yoga | Think or write about 3 things you are grateful for | Take planned breaks | Stick to consistent sleep/wake times |
| Go to bed earlier by __ mins | Spend time in nature | Meditate/Practice mindfulness | Tackle a task on your to do list | Express your gratitude to someone you care about |
| Turn of screens __ mins before bed | Spend time in the sun | Spend time in the sun | Switch tasks when you get stuck | Put your phone on ‘Do No Disturb’ |
| Read or listen to a podcast | Exercise lightly (walk, dance) | Treat yourself to a delicious snack or drink | Clean and organize your working space | Routinely visit a public space (coffee shop, park, farmer’s market) |
| Listen to relaxing music | Take a restorative shower or bath | Listen to nature sounds or white noise | Change into appropriate clothes | Tell someone your worries/express your feelings |
| Take some deep breaths | Take some deep breaths | Confide in a friend and express your feelings | Schedule something you’ve been putting off | Meditate/practice mindfulness |
| Avoid large meals | Take a power nap (about an hour) | Take some deep breaths | Drink plenty of water | Eat a healthy balanced meal |
| Practice yoga/stretch your body | Listen to music while exercising | Clean and organize your space | Listen to music while working | Confide in a friend and express your feelings |
| Make your bed | Exercise intensely (HIIT, sprints, jump rope) | Change into comfortable clothing | Eat a healthy, balanced meal | Limit social media use |
| Pray/read religious texts | Meditate/practice mindfulness | Text/call a loved one or friend | Exercise moderately (run, bike, lift weights) | Find opportunities to mentor, coach, or teach others |
| Exercise lightly (walk, dance) | Stretch | Pray/read religious texts | Spend time in nature | Plan a vacation with loved ones or friends |
| Visualize your happy place | Treat yourself to a delicious snack or drink | Take a restorative shower or bath | Take some deep breaths | Talk to or seek out a mental health professional |
| Spend time in nature | Take breaks from sitting | Eat a healthy, balanced meal | Meditate/practice mindfulness | Reach out to someone you haven’t talked to in a while |
| Eat a healthy, balanced meal | Go to bed earlier by __ mins | Practice yoga/stretch your body | Spend time in the sun | Plan free time in your schedule |
| Spend time in the sun | Pray/read religious texts | Drink plenty of water | Exercise lightly (walk, stretch, dance) | Join an online community (Facebook groups, virtual book clubs) |
